# Recent advances in Polygonaceae endophytes: diversity, secondary metabolites and biotechnological applications

**DOI:** 10.3389/fbioe.2025.1666908

**Published:** 2025-09-26

**Authors:** Zhi-Min Chen, Rui-Qi Tang

**Affiliations:** Key Laboratory of Natural Microbial Drugs Research of Jiangxi Province, Key Laboratory of Microbial resources and metabolism of Nanchang City, College of Life Sciences, Jiangxi Science and Technology Normal University, Nanchang, Jiangxi, China

**Keywords:** Polygonaceae, endophytes, secondary metabolites, growth-promoting, stress tolerance, bioactivities

## Abstract

The Polygonaceae family comprises numerous traditional Chinese medicinal herbs and serves as a significant source of natural products with bioactive properties. Endophytes, which colonize the internal tissues of plants, have various beneficial effects on their hosts. The diverse communities of endophytes within Polygonaceae plants can promote host growth and enhance stress resistance by producing plant hormones and improving the metabolic levels of host cells. Additionally, endophytes can induce the accumulation of secondary metabolites in host plants. Furthermore, endophytes possess the capability to produce a variety of bioactive compounds, which can be further utilized in the biosynthesis of natural pharmaceuticals. Although research on endophytes of Polygonaceae plants has made notable progress, it has been rarely reviewed comprehensively. This review focuses on the diversity of endophytes and their effects on host plants in four representative genera of Chinese medicinal Polygonaceae: *Reynoutria*, *Fagopyrum*, *Rheum*, and *Rumex*. We also discuss the application of bioactive metabolites produced by these endophytes and summarize recent advances in their biosynthesis in microbial cell factories. The review aims to provide insights into the identification and application of endophytic microbial resources from Polygonaceae plants.

## 1 Introduction

The Polygonaceae family, a prominent taxonomic group of medicinal plants primarily found in the temperate zones of northern hemisphere, comprises approximately 1,200 species systematically classified into 46 genera (Zhang et al., 2022a). Several species within the Polygonaceae family, including *Rheum palmatum*, *Reynoutria japonica* (also known as *Fallopia japonica* or *Polygonum cuspidatum*), and *Fagopyrum dibotrys*, are recognized as traditional Chinese medicinal herbs or ethnopharmacological resources, with these representative species officially listed in the Pharmacopoeia of the People’s Republic of China: Volume I (National Pharmacopoeia Commission, 2020). Currently, a range of bioactive compounds have been identified from Polygonaceae plants, such as emodin, polydatin, resveratrol, and quercetin, which exhibit antioxidant, antibacterial, anti-inflammatory, and anticancer activities (Vasas et al., 2015; Jing et al., 2016; Mitra et al., 2022; Ke et al., 2023). In recent years, the continuous expansion of the global traditional Chinese medicine market has led to dramatic declines in wild populations of many medicinal Polygonaceae plants due to unsustainable harvesting practices and ecosystem degradation. The traditional medicinal herb *F. dibotrys* has been officially included in the *List of National Key Protected Wild Plants*. Additionally, several *Rheum* species have been listed in the *China Biodiversity Red List*—*Higher Plants Volume*, with *Rheum globulosum* classified as endangered and *Rheum subacaule* designated a critically endangered. Therefore, it is urgent to find a balance between the conservation and utilization of plant resources. Fortunately, plant microbial communities, such as endophytes, have been extensively studied for their capacity to produce secondary metabolites, making them crucial for the conservation of plant resources and the production of natural medicines (Yan et al., 2019; Gupta et al., 2020).

Plant endophytes, primarily bacteria and fungi, are microorganisms that colonize within plant tissues without causing apparent disease symptoms in the host plant (Tiwari et al., 2023). Research has demonstrated that plant endophytes can enhance the growth and stress tolerance of host plants, stimulate the accumulation of secondary metabolites, and have the potential to be developed as biocontrol agents (Li et al., 2023; Watts et al., 2023; Gowtham et al., 2024; Qin et al., 2024). Furthermore, plant endophytes represent a promising source of novel bioactive compounds, which have significant biotechnological potential in sustainable agriculture and pharmacognosy (Nazir et al., 2024; Zotchev, 2024). Therefore, screening endophytes from Polygonaceae plants that can enhance the growth and quality of host plants or produce medicinal active compounds is crucial for the conservation of Polygonaceae plants and the pharmaceutical industry. Previously, the diversity of endophytes in *Rumex* plants and the pharmacological activities of their metabolites have been reviewed (Ntemafack et al., 2023). However, it lacks an introduction to the biosynthetic pathways of the metabolites and the discussion of the effects of endophytes on their host plants. Notably, significant progress has been made on endophytes in many Polygonaceae plants, particularly in the representative medicinal genera *Reynoutria*, *Rheum*, and *Fagopyrum*, yet the progress has not been comprehensively summarized. This review focuses on the endophytes in plants from the genera *Reynoutria*, *Fagopyrum*, *Rheum*, and *Rumex* of the Polygonaceae family, summarizing their diversity, effects on host plants, biological activities of their secondary metabolites, and the microbial biosynthesis of these metabolites.

## 2 Diversity of endophytes in medicinal Polygonaceae plants

Within the family Polygonaceae, culturable and non-culturable endophytic communities from various genera have been investigated using microbial culture-based methods and high-throughput sequencing techniques. The diversity of endophytic communities is influenced by plant age, season, growth environments, and the specific plant tissues colonized by endophytes. Here, we review the findings regarding endophytic fungi (Table 1) and bacteria (Table 2) of four genera within the Polygonaceae family, *Reynoutria*, *Fagopyrum*, *Rumex*, *Rheum*.

**TABLE 1:** Culturable endophytic fungi of Polygonaceae plants.

Host species	Tissue of isolation	Fungal genus	Reference
*Reynoutria japonica*	Root	*Cladosporium, Penicillium*	Liu et al. (2020b)
*Reynoutria japonica*	Root	*Aspergillus, Cladosporium, Cunninghamella, Fusarium, Paecilomyces, Penicillium, Termitomyces, Trematosphaeria, Trichoderma*	Xu et al. (2020)
*Reynoutria japonica*	Root, stem	*Alternaria, Cephalosporium, Geotrichum, Mucor*	Shi et al. (2012)
*Reynoutria japonica*	Leaf	*Alternaria, Colletotrichum, Pestalotiopsis, Phoma, Phomopsis*	Kurose et al. (2012)
*Fagopyrum dibotrys*	Root, stem, leaf	*Alternaria, Colletotrichum, Fusarium*	Xie et al. (2024)
*Fagopyrum esculentum*	Seed	*Alternaria, Aureobasidium, Botryotinia, Epicoccum, Fusarium, Stereum*	Kovačec et al. (2016)
*Rheum palmatum*	Root	*Fusarium*	You et al. (2013)
*Rheum officinale*	Root	*Mucor*	Zhang et al. (2018)
*Rheum spiciforme*	Leaf	*Aureobasidium, Fusarium*	Khan et al. (2023)
*Rumex acetosa*	Root, leaf	*Acremonium, Alternaria, Arthrinium, Aspergillus, Aureobasidium, Cladosporium, Clonostachys, Coniothyrium, Cylindrocarpon, Fusarium, Geniculosporium, Gonatobotrys, Helicosporium, Humicola, Microspheropsis, Mucor, Penicillium, Phoma, Septofusidium, Sterile, Trichocladium, Trichoderma, Zygorhynchus*	Wearn et al. (2012)
*Rumex acetosa*	Pollen, seed, leaf	*Acremonium, Alternaria, Aspergillus, Cladosporium, Epicoccum, Fusarium, Mucor, Phialophora, Tricothecium*	Hodgson et al. (2014)
*Rumex hastatus*	Root	*Anguillospora, Beltrania, Cylindrocarpon, Helicosporium, Seiridium, Setosynnema*	Sati and Pathak (2017)
*Rumex nervosus*	Leaf	*Penicillium*	Hassane et al. (2022)
*Rumex madaio*	Root, leaf	*Colletotrichum, Fusarium*	Bai et al. (2019)

**TABLE 2:** Culturable endophytic bacteria of Polygonaceae plants.

Host species	Tissue of isolation	Bacterial genus	Reference
*Reynoutria japonica*	Root	*Bacillus*	Liu et al. (2020b)
*Reynoutria japonica*	Root	*Streptomyces*	Wang et al. (2016)
*Rumex acetosa*	Root, stem	*Arthrobacter*, *Bacillus*, *Curtobacterium, Enterobacter*, *Nocardioides*, *Pantoea*, *Plantibacter*, *Pseudomonas*, *Rhizobium*	He et al. (2018)
*Rumex acetosa*	Root, stem, leaf	*Microbacterium, Plantibacter, Pseudomonas*	Burges et al. (2017)
*Rumex acetosa*	Leaf	*Bacillus*	Zhang et al. (2023)
*Rumex acetosa*	Root	*Bacillus*	Xu et al. (2022)
*Rumex dentatus*	Leaf	*Bacillus*	Ntemafack et al. (2022b)
*Rumex dentatus*	Root, stem	*Streptomyces*	Qiu et al. (2015)
*Rumex dentatus*	Root	*Streptomyces*	Ntemafack et al. (2022a)

### 2.1 Diversity of endophytic fungi in Polygonaceae

Fungal endophytes are widely distributed in plants and exhibit significant diversity in species (Hodgson et al., 2014). Within the genus *Reynoutria*, *R*. *japonica* is known for its adaptability and medicinal properties, making it a subject of extensive research (Ke et al., 2023). The root, stem, and leaf of *R*. *japonica* are commonly used in endophyte studies. Seventeen fungal genera were identified, in which, *Alternaria*, *Cladosporium* and *Penicillium*, were identified twice in different studies (Table 1). Plants of the genus *Fagopyrum* have a long history of medicinal and edible applications and possess considerable medicinal potential (Jing et al., 2016). Endophytic fungi from three genera were isolated from the roots, stems, and leaves of *F*. *dibotrys*, while six genera were identified from the seeds of *Fagopyrum esculentum* (Kovačec et al., 2016; Xie et al., 2024). The strains isolated in both studies belong to the genera *Alternaria* and *Fusarium*. Plants in the genus *Rheum* are widely recognized for their medicinal properties (Zhuang et al., 2020). However, the number of endophytes isolated from *Rheum* is relatively low, *Fusarium* and *Mucor* were identified from the roots of *R. palmatum* and *Rheum officinale*, respectively (You et al., 2013; Zhang et al., 2018). Two fungal genera *Aureobasidium* and *Fusarium* were identified from the leaves of *Rheum spiciforme* (Khan et al., 2023). The genus *Rumex* encompasses numerous medicinal plants, and studies on endophytes in *Rumex* are prevalent than those in the other three genera (Ntemafack et al., 2023). The diversity of endophytic fungi in *Rumex acetosa* has been shown to be high, with strains belonging to 23 genera isolated from the roots and leaves (Wearn et al., 2012). In another study, nine genera were identified from pollen, seeds and leaves, six of which overlapped with the previous study (Hodgson et al., 2014). Endophytes in other species of *Rumex*, such as *Rumex hastatus*, *Rumex nervosus* and *Rumex madaio*, have also been investigated. Six genera were identified from the roots of *R. hastatus*, with four genera being unique to this species, apart from *Cylindrocarpon* and *Helicosporium* (Sati and Pathak, 2017). *Penicillium* was identified in the leaves of *R. nervosus* (Hassane et al., 2022), while *Colletotrichum* and *Fusarium* were detected in both the roots and leaves of *R. madaio* (Bai et al., 2019).

As shown in Table 1, *Fusarium* emerges as the most prevalent genus of endophytic fungi within the culturable range, identified across many species of the four genera. *Alternaria*, *Aureobasidium*, *Colletotrichum*, and *Mucor* were found in three genera. *Aspergillus*, *Cladosporium*, *Phoma*, and *Penicillium* were identified in *Reynoutria* and *Rumex*, while *Epicoccum* was identified in both *Fagopyrum* and *Rumex*. Although limited studies have been conducted on the endophytes of *Fagopyrum* plants, two unique fungal genera, *Stereum* and *Botryotinia*, were identified within this genus.

Culture-independent methods, such as high-throughput sequencing, enable researchers to gain a more comprehensive understanding of the plant endophytic community. The diversity of endophytes can be influenced by the tissues and ages of host plants. Aleynova et al. (2024) investigated the diversity of endophytic fungi in the roots, stems, leaves, flowers and seeds of *R. japonica* using next-generation sequencing. The root, stem, and leaf exhibited greater diversity of endophytic fungi compared to the flower and seed. Furthermore, the relative abundance of endophytic fungi varied across different tissues of *R. japonica*. For example, *Alternaria* was the dominant genus in seeds, while *Microcyclosporella* was predominant in stems and flowers, with relative abundances of 18.11% and 50.18%, respectively (Aleynova et al., 2024). Chen et al. (2023) investigated the endophyte diversity of *R. palmatum* across different tissues, namely, root, stem, and leaf, as well as different plant ages, specifically 1-, 2-, and 3-year-old plants. They found that the dominant genera in root, stem, and leaf were *Dactylonectria* (71.95%), *Cladosporium* (39.56%), and *Russula* (25.54%), respectively. Notably, *Dactylonectria* was the predominant genus across samples of different ages, although its relative abundance decreased from 91.49% to 3.43% as the age of the plants increased (Chen et al., 2023). External factors, such as the cultivation area of the host plant and the season in which the samples were collected, can also influence endophytic microbial communities. Zhong et al. (2024) identified 119 genera of endophytic fungi from the seeds of *F*. *tataricum* and *F. esculentum* using a high-throughput sequencing method, with *Alternaria* being the dominant genus, exhibiting over 50% relative abundance in every sample. Other highly abundant genera included *Botrytis*, *Cladosporium*, *Epicocum*, *Filobasidium*, and *Stemphylium* (Zhong et al., 2024). Conversely, another study reported that *Cryptococcus*, *Aureobasidium*, *Botrytis*, *Acremonium*, and *Didymella* were the dominant genera in the seeds of *F*. *esculentum* (Li et al., 2021). In *R. palmatum*, endophytic fungi belonging to 265 genera were identified, with the dominant genera being *Dactylonectria*, *Clonostachys*, *Leptosphaeria*, *Chaetomium*, *Fusarium*, and *Aspergillus* across different geographical areas (Chen et al., 2021). The diversity of endophytic fungi in the roots of *R*. *palmatum* varied across the seasons, with *Phialophora* dominating in Spring and Summer, and *Nothodactylaria* in Autumn (Li et al., 2024). In addition, anthropogenic factors also affect the endophytes communities, for example, low-pressure cold plasma treatment has been shown to alter the fungal community structure in *Fagopyrum* (Mravlje et al., 2021).

### 2.2 Diversity of endophytic bacteria in Polygonaceae

Endophytic bacteria are widely colonized in plants, and many have garnered considerable attention due to their plant growth-promoting properties (Santoyo et al., 2016). Compared to endophytic fungi in Polygonaceae plants, endophytic bacteria have been studied less frequently, yet they still exhibit notable diversity. Within the culturable range, *Bacillus* and *Streptomyces* are commonly found in the genera *Reynoutria* and *Rumex*. As shown in Table 2, only one genus, either *Bacillus* or *Streptomyces* was identified in most studies. Burges et al. (2017) isolated strains belonging to *Microbacterium*, *Plantibacter*, and *Pseudomonas* from the roots, stems, and leaves of *R. acetosa*. No strains belonging to *Bacillus* or *Streptomyces* were reported in this study, which may be attributed to the goal of isolating strains with plant growth-promoting activities. In another study, strains from nine bacterial genera were identified from the roots and stems of *R. acetosa* (He et al., 2018).

In addition to culture-based methods, high-throughput sequencing was employed to study the diversity of endophytic bacteria in Polygonaceae plants. The endophyte diversity among different *R*. *palmatum* samples was comprehensively analyzed. The dominant bacterial phylum in all samples was Proteobacteria, while at the genus level, the dominant genus varied across different areas (Chen et al., 2021), tissues and ages (Chen et al., 2023), as well as seasons (Li et al., 2024). For instance, in samples from 2-year-old *R. palmatum*, the dominant bacterial genera of roots, stems and leaves were *Microbacterium*, *Rahnella*, and *Methylobacterium*, respectively (Chen et al., 2023). The relative abundance of the dominant bacterial genera of *R. palmatum* gradually increased from Spring to Autumn (Li et al., 2024).

In summary, the composition and diversity of endophytic communities in Polygonaceae plants are significantly influenced by regional environments, seasonal variations, host plant tissues, and plant ages. The root tissue is the most commonly studied among *Reynoutria*, *Fagopyrum*, *Rheum*, and *Rumex*, followed by leaf and stem tissues. While seeds and pollen have been used to isolate endophytic fungi, reports on endophytic bacteria in these two tissues of Polygonaceae plants remain scarce. Among the endophytes isolated from these plants, fungi constitute the majority, whereas bacteria are considerably less abundant. Endophytic fungi from the genera *Fusarium* and *Alternaria* are the most frequently isolated, with strains from *Cladosporium*, *Mucor* and *Penicillium* also being frequently isolated. *Bacillus* and *Streptomyces* are the most frequently reported genera of endophytic bacteria. Furthermore, studies have demonstrated significant correlations between the endophyte diversity, particularly endophytic fungi, and the accumulation of bioactive compounds in Polygonaceae species (Chen et al., 2021; Li et al., 2024; Zhong et al., 2024). In addition, endophytes associated with Polygonaceae plants exhibit growth-promoting potential in their hosts (Qu et al., 2023; Chen et al., 2024). Therefore, Polygonaceae plants harbor diverse endophytic communities, rendering them valuable systems for investigating the mechanisms of host-microbe interactions.

## 3 Effects of endophytes on Polygonaceae plants

**FIGURE 1:**
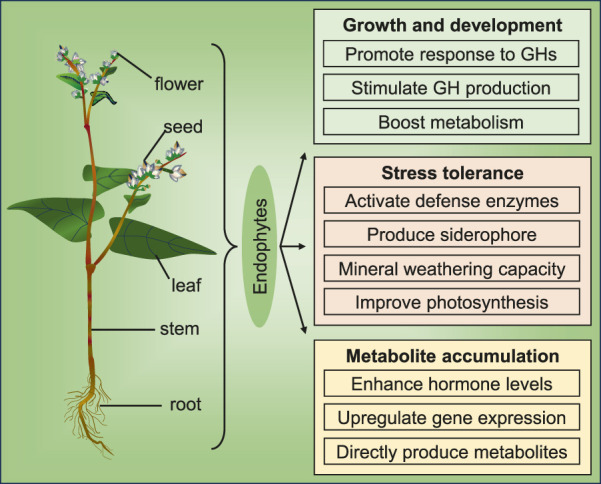
An overview of the effects of endophytes on Polygonaceae plants (GH: growth hormone).

Endophytes reside within plants and influence their growth, development, and metabolism (Yan et al., 2019; Mushtaq et al., 2022; Qin et al., 2024). Endophytes associated with the Polygonaceae family exhibit a variety of beneficial effects on host plants, including the promotion of growth and development, enhancement of stress tolerance, and stimulation of secondary metabolite accumulation (Figure 1). In recent years, researchers have increasingly investigated the mechanisms underlying the effects of endophytes on their hosts.

### 3.1 Promotion of growth and development

Chemical fertilizers play a crucial role in enhancing crop yields, however, their excessive use raises significant environmental concerns (Atieno et al., 2020). The development and application of biofertilizers are regarded as viable methods to mitigate environmental pollution. Endophytes, in particular, present promising biofertilizer options due to their beneficial effects on plant growth. Research has demonstrated that endophytes from Polygonaceae plants can promote host growth, with the underlying mechanisms being primarily investigated. For instance, the endophytic fungus *Trichoderma citrinoviride* HT-1 has been shown to enhance the growth of *R. palmatum* by upregulating the expression of the host’s Gretchen Hagen 3 (GH3) and ethylene response factor (ERF) genes, thereby improving the host’s response to auxin and ethylene (Chen et al., 2022b). Similarly, *Plectosphaerella cucumerina* J-G upregulated the expression of hormone-responsive genes in *Rumex gmelinii*, further enhancing the host’s response to auxin and cytokinin (Ding et al., 2022). In addition, the expression of genes involved in amino acid metabolism and carbohydrate synthesis in *R*. *gmelinii* was enhanced by *P*. *cucumerina* J-G, thereby increasing the substrate and energy pool available to the host. Furthermore, colonization by *Serendipita indica* led to an increase in indole-3-acetic acid (IAA) content in *F*. *tataricum* by upregulating the expression of genes encoding key enzymes in the indole-3-pyruvic acid (IPyA) pathway (Zheng et al., 2023). Beyond the strains themselves, treating *F*. *tataricum* sprouts with mycelial extracts and polysaccharides from endophytic fungi as growth inducers has also been shown to promote their growth (Zhao et al., 2014; Zhong et al., 2016).

### 3.2 Enhancement of stress tolerance

The cultivation of plants faces challenges such as low soil fertility, drought, and salt stress (Nawaz et al., 2023). Additionally, plant diseases caused by pathogens can adversely affect the growth, development, and quality of plants (Park et al., 2019). Fortunately, studies have shown that endophytes enhance host stress tolerance against both environmental and biotic factors (Watts et al., 2023; Gowtham et al., 2024). Inoculation of endophytic bacteria belonging to the genera *Pseudomonas*, *Microbacterium*, and *Plantibacter* significantly increased the contents of chlorophylls and carotenoids in *R. acetosa*, thereby reducing the stress level of the plant (Burges et al., 2017). Furthermore, this endophyte inoculation enhanced the activity of acid phosphatase in the soil microbial communities associated with *R. acetosa*, thereby improving nutrient cycling. Similarly, endophytic fungi such as *Bionectria* sp. Fat6 and *S*. *indica* from *F*. *tataricum* demonstrated the ability to increase the chlorophyll content of the host (Xiang et al., 2021; Zheng et al., 2023). In another study, endophytic bacteria exhibiting efficient siderophore production and mineral weathering capabilities were isolated from *R*. *acetosa*, which may assist the host plant in adapting to nutrient-deficient and rocky soil environments (He et al., 2018). Transcriptomic analysis indicated that the inoculation of endophytic fungi in *R*. *gmelinii* upregulated the expression of gene encoding phenylalanine ammonia lyase (PAL), a key enzyme in the synthesis of secondary metabolites involved in stress resistance, thereby enhancing the host’s resistance (Ding et al., 2022).

### 3.3 Stimulation of secondary metabolite accumulation

Endophytes can influence the accumulation of host metabolites by regulating the metabolic pathway of hosts or synthesizing the metabolites (Yang et al., 2016; Xu et al., 2023). Endophytes have been shown to enhance the accumulation of anthraquinones in *R*. *palmatum* by upregulating genes that encode key enzymes in the polyketide pathway, including acetolactate synthase, chalcone synthase (CHS), and beta-amyrin synthase (Chen et al., 2022b). Similarly, endophytes derived from *R. gmelinii* promoted the accumulation of resveratrol and polydatin in the host by upregulating the expression of the PAL gene while downregulating the expression of cinnamoyl-CoA reductase and shikimic acid O-hydroxycinnamoyl transferase genes (Ding et al., 2022). Furthermore, the mycelial polysaccharides from endophytes associated with *F. tataricum* stimulated the phenylpropanoid pathway in the host, thereby increasing flavonoid content (Zhong et al., 2016). Endophytes can also enhance the accumulation of secondary metabolites in the host by promoting the production of plant hormones. For instance, the endophytic fungus *S*. *indica* can stimulate the biosynthesis of anthocyanins in *F*. *tataricum* by elevating the levels of jasmonic acid and abscisic acid in the host (Zheng et al., 2023). Additionally, endophytes can directly regulate the chemical composition of plants through biosynthesis. Secondary metabolites commonly found in Polygonaceae plants, such as emodin, rutin, and resveratrol, can be synthesized directly by endophytes (Shi et al., 2012; You et al., 2013; Hassane et al., 2022). Moreover, endophytes possess the capability to convert polydatin, which is abundant in *R*. *japonica*, into resveratrol (Liu et al., 2020b). The microbial production of these secondary metabolites significantly contributes to their accumulation within the host.

In summary, endophytes in Polygonaceae plants promote host growth by enhancing the response to growth hormones and auxin synthesis, as well as the increasing the metabolic levels of plant cells. These endophytes enhance the adaptability of Polygonaceae plants to stress conditions. Furthermore, endophytes from Polygonaceae plants have potential applications in crop cultivation. For instance, the endophytic *Streptomyces* isolated from *R*. *dentatus* has been shown to effectively promote the growth of rice (Ntemafack et al., 2022a). Similarly, the endophytic *Bacillus* from *R*. *dentatus* can mitigate the damage caused by the pathogenic *Fusarium oxysporum* to potato tubers (Ntemafack et al., 2022b). Endophytes facilitate the accumulation of secondary metabolites in Polygonaceae plants by enhancing the expression of genes encoding key enzymes in the synthetic pathways of these metabolites or by directly producing the metabolites themselves. The ability of endophytes to increase the metabolite content in hosts and synthesize metabolites identical to those of their hosts offers viable strategies for enhancing the production of bioactive compounds from Polygonaceae species. For example, co-culturing the host with endophytes to promote metabolite production, and heterologously expressing the synthetic pathway of endophyte metabolites in microbial cell factories are effective strategies, which are beneficial for the sustainability of medicinal Polygonaceae plants. Moreover, endophytes produce novel bioactive metabolites with medicinal value, and investigating their metabolic products represents a promising avenue for natural drug discovery.

## 4 Biological activity of Polygonaceae endophytes and their secondary metabolites

**FIGURE 2:**
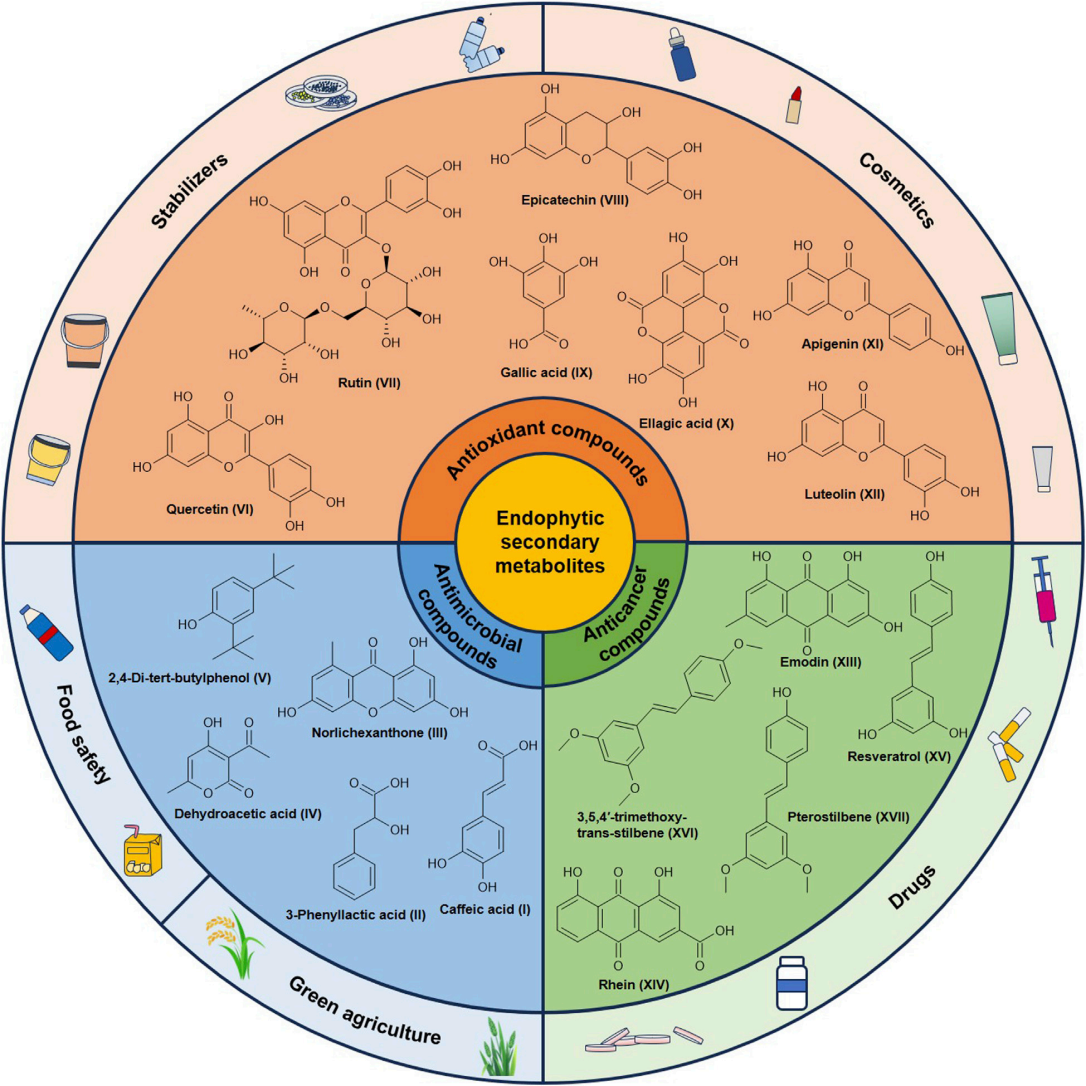
Secondary metabolites of endophytes from Polygonaceae plants and their applications.

Medicinal plants are critical sources for the development of novel therapeutic agents due to their abundance of natural bioactive compounds (Gómez and Luiz, 2018). However, the yield and quality of these plants are susceptible to environmental factors. Additionally, the extraction efficiency of bioactive compounds remains suboptimal (Milke et al., 2018; Abo-Kadoum et al., 2022). Endophytes of plants produce diverse secondary metabolites that exhibit biological activities comparable to those derived from their host plants, acting as reservoirs of natural bioactive compounds (Ancheeva et al., 2020). Secondary metabolites of endophytes from Polygonaceae plants demonstrate potential in anticancer, antioxidant, and antimicrobial applications (Figure 2).

### 4.1 Antimicrobial activity of endophytes

Root rot, caused by plant pathogens such as *F*. *oxysporum*, significantly reduces crop yields and compromises the quality of medicinal plants (Pandey et al., 2021). Endophytes exhibit antagonistic capabilities against these pathogens (Pal et al., 2021), and various secondary metabolites produced by them exhibit anti-pathogenic activity (Waqar et al., 2024). The ethyl acetate extract of *Alternaria alstroemeriae*, an endophyte derived from *F. dibotrys*, contains antimicrobial compounds including caffeic acid (I), 3-phenyllactic acid (II), and norlichexanthone (III) (Xie et al., 2024). Caffeic acid inhibits pathogenic microorganisms by disrupting the integrity of cell membranes and hindering mycelial growth (Khan et al., 2021). Both 3-phenyllactic acid and norlichexanthone can inhibit the expression of bacterial virulence factors and the biofilm formation by competitively binding to quorum-sensing factors in bacteria (Baldry et al., 2016; Wu et al., 2023). The endophyte *Streptomyces* sp. A0916 from *R*. *japonica* produced the antibacterial substance dehydroacetic acid (IV) (Wang et al., 2016), which exerts inhibitory effect on fungi such as *Botrytis cinerea* and *Sclerotinia sclerotiorum* (Huang et al., 2024). The endophytic bacterium *Bacillus* sp. KL5 from *R*. *dentatus* and the fungus *T. citrinoviride* HT-1 from *R*. *palmatum* have demonstrated inhibitory activity against the common plant pathogen *F. oxysporum* (Chen et al., 2022a; Ntemafack et al., 2022b). *Bacillus* sp. KL5 can produce 2,4-di-tert-butylphenol (V), an effective antibacterial agent that capable of inhibiting pathogenic fungi (Ntemafack et al., 2022b).

### 4.2 Antioxidant activity of endophytes

Endophytes are promising sources of antioxidant compounds, particularly polyphenols and flavonoids (Rai et al., 2021; Shen et al., 2022). Quercetin (VI), a flavonoid known for its exceptional antioxidant capacity, is commonly found in plants belonging to the Polygonaceae family. Xie et al. (2024) reported that the endophytic fungus *A. alstroemeriae* isolated from *F. dibotrys*, exhibited the ability to produce quercetin. Numerous common secondary metabolites in plants, such as rutin (VII), epicatechin (VIII), gallic acid (IX), ellagic acid (X), apigenin (XI), and luteolin (XII), have been shown to be produced by *Penicillium* isolated from *R*. *nervosus* (Hassane et al., 2022). Among these compounds, rutin, epicatechin, apigenin, and luteolin are flavonoids that have demonstrated potent antioxidant properties (Shen et al., 2022).

### 4.3 Anticancer activity of endophytes

Endophytes can produce a variety of anticancer compounds. Emodin (XIII), an anthraquinone, has demonstrated promising anticancer properties against various cancers and has been reported to be produced by two endophytic fungi *Fusarium solani* and *Polyporales* sp. Isolated from plants of the genus *Rheum* (You et al., 2013; Dar et al., 2017). Moreover, *F. solani* is capable of producing another anthraquinone compound rhein (XIV), which also exhibits significant anticancer activity (You et al., 2013). Additionally, resveratrol (XV), an effective anticancer compound against various tumor cell types, was produced by *Alternaria* sp. HG6 isolated from *R. japonica* (Shi et al., 2012). Furthermore, endophytes from *R. japonica* have been shown to transform resveratrol into its derivatives which exhibit improved stability and bioavailability. Specifically, *Streptomyces* sp. A12 and *Penicillium* sp. F5 transformed resveratrol into 3,5,4′-trimethoxy-trans-stilbene (XVI) and pterostilbene (XVII), respectively (Tian et al., 2018; Xu et al., 2020).

In summary, endophytes and their metabolites in Polygonaceae plants exhibit a broad spectrum of antimicrobial activity, effectively inhibiting several common plant pathogens and showing promise as biological control agents. Furthermore, like their hosts, endophytes can synthesize phenolic compounds and flavonoids, positioning them as a potential source of antioxidant substances. Additionally, certain metabolites produced by endophytes in Polygonaceae exhibit anticancer activity, capable of directly inhibiting tumor cell proliferation or aiding chemotherapeutic drugs in cancer treatment. The potential of endophytes and their metabolites in Polygonaceae plants extends to both agricultural cultivation and drug development. Investigating the functional characteristics of these endophytes and pursuing their translational applications in agricultural and pharmaceutical fields are important future research directions, that will promote the conservation of medicinal plants and advance the natural medicine industry.

## 5 Biosynthesis of metabolites of endophytes from Polygonaceae plants

Various metabolites produced by endophytes from Polygonaceae plants are also common natural products extensively studied for their broad-spectrum biological activities. To address the challenge of low yield and efficiency in plant extraction, microbial synthesis of these metabolites has received increasing attention. Here, we provide a concise overview of the key enzymes involved in biosynthetic pathways of these metabolites and their heterologous synthesis in microorganisms.

### 5.1 Biosynthesis of resveratrol

Resveratrol is a significant medicinal component found in *R*. *japonica* and other plants such as grapes and peanuts. Currently, plant extraction is the main source of commercial resveratrol, however, this method is highly dependent on the availability of plants and is often inefficient. Consequently, microorganisms have been employed to develop synthetic methods for resveratrol production. The key enzymes involved in the resveratrol synthetic pathway include PAL/tyrosine ammonia lyase (TAL), trans-cinnamate 4-hydroxylase (C4H), 4-coumarate-CoA ligase (4CL), stilbene synthase (STS), and resveratrol synthase (ST) or CHS (Abo-Kadoum et al., 2022). The microbial production of resveratrol was first reported 2 decades ago, where *Saccharomyces cerevisiae* was used as a heterologous host for the expression of plant-derived 4CL and ST, resulting in the production of 1.45 μg/L of resveratrol glucoside (Becker et al., 2003). Since then, the microbial synthesis of resveratrol has been extensively studied and reported. In addition to the conventional yeast *S*. *cerevisiae*, non-conventional yeasts such as *Yarrowia lipolytica* and bacteria such as *Escherichia coli* have also been utilized as hosts. Currently, the titers of resveratrol achieved are 2.3 g/L, 4.1 g/L, and 22.5 g/L in the three commonly used hosts *E. coli*, *S*. *cerevisiae*, and *Y*. *lipolytica*, respectively (Lim et al., 2011; Liu et al., 2022; Meng, et al., 2023). Lim et al. (2011) enhanced resveratrol production by optimizing combinations of 4CL- and STS-encoding genes from various sources, selecting appropriate promoters for gene expression, and improving strain backgrounds, as well as enhancing the intracellular supply of precursors. Meng et al. (2023) reported the expression of genes encoding 4CL, STS, and a bi-functional PAL/TAL in a *S. cerevisiae* strain, enabling the strain to synthesize resveratrol using yeast extract peptone dextrose (YPD) medium. By further increasing the copy number of pathway genes, enhancing precursor supply, and tailoring the engineered strain, a resveratrol titer of 4.1 g/L was achieved using minimal medium through fed-batch fermentation. Liu et al. (2022) constructed a basal resveratrol-producing strain by expressing TAL, 4CL, and STS genes in *Y*. *lipolytica*. The resveratrol titer was subsequently enhanced through multiple strategies, including optimization of enzyme gene sources and copy numbers, enhancement of precursor supply, and control of cell morphology via process engineering. While the aforementioned studies utilized genes from plants, Lu et al. (2021) identified genes encoding 4CL and CHS from a resveratrol-producing endophytic fungus derived from grape, whose expression enabled *S. cerevisiae* to produce *p*-coumaroyl CoA and resveratrol, respectively. Shi et al. (2012) isolated several resveratrol-producing endophytic fungi from *R*. *japonica*, however, the genetic information related to resveratrol synthesis remains unexplored.

### 5.2 Biosynthesis of emodin

Emodin is an anthraquinone compound synthesized through the polyketide pathway, a metabolic route shared by bacteria, fungi, and plants (Mund and Čellárová, 2023). It is primarily isolated from Polygonaceae plants, particularly *R*. *japonica* (Zhang et al., 2022b). However, the extraction process necessitates substantial quantities of plant materials and is inefficient. Consequently, research has shifted towards the microbial synthesis of emodin. Acetyl CoA and malonyl CoA serve as precursors for the biosynthesis of emodin, requiring various enzymes, including acetyl CoA carboxylase (ACC1), polyketide synthase (PKS), metallo-β-lactamase-type thiesterase (MβL-TE), and decarboxylase (DC) (Liu et al., 2020a; Zhang et al., 2022b). Endophytic fungi from *R*. *palmatum* and *Rheum emodi* have been reported to possess the capability of producing emodin (You et al., 2013; Dar et al., 2017). However, the key enzymes involved in their synthetic pathway remain to be elucidated. Genes encoding enzymes related to the emodin synthetic pathway in other fungi have been investigated and expressed in *S*. *cerevisiae*. Sun et al. (2019) expressed a non-reducing PKS alongside MβL-TE in *S*. *cerevisiae*, while simultaneously introducing DC, resulting in an engineered strain with an emodin yield of 96.5 mg/L. Furthermore, the expression of ACC1 has been shown to increase the content of malonyl-CoA. Thus, a double-point mutant ACC1^S659A, S1157A^ was introduced into the engineered strain to further enhance emodin yield, resulting a strain with an emodin yield of 253.2 mg/L (Sun et al., 2019). Biosynthetic technology presents significant potential for the heterologous synthesis of emodin, further exploration of the genes within its biosynthetic pathway is necessary.

### 5.3 Biosynthesis of flavonoids

Flavonoids are prevalent bioactive secondary metabolites found in plants of Polygonaceae family, as well as an important group of metabolites produced by their endophytes (Hassane et al., 2022; Li et al., 2022; Zou et al., 2023; Xie et al., 2024). Endophytes associated with Polygonaceae plants have been reported to synthesize various flavonoids, including quercetin, luteolin, apigenin, rutin, and epicatechin. Traditional methods for flavonoid production, such as plant extraction and chemical synthesis, have proven inefficient for large-scale industrial applications (Tariq et al., 2023). Consequently, there has been growing interest in the genetic engineering of microorganisms for scalable flavonoid production (Sheng et al., 2020). Naringenin and eriodictyol serve as key precursor molecules in the biosynthesis of various flavonoids, utilizing tyrosine as a substrate, with the process involving the enzymes TAL, 4CL, CHS and chalcone isomerase (CHI) (Dunstan et al., 2020). Yiakoumetti et al. (2023) expressed type II FNS (FNS-II) from *Lonicera japonica* and cytochrome P450 reductase (CPR) from *Arabidopsis thaliana* (AtCPR) in a naringenin-producing *E*. *coli*, achieving apigenin yield of 128 mg/L. Furthermore, the expression of FNS-II from *Glycine max* and AtCPR in an eriodictyol-producing *E*. *coli* resulted in a strain that produced 5.0 mg/L luteolin (Yiakoumetti et al., 2023). Notably, the source of FNS-II significantly influenced the yields of apigenin and luteolin. In another study, Rodriguez et al. (2017) constructed an engineered *E*. *coli* strain that produced 20.38 mg/L quercetin by overexpressing 4CL, CHS, CHI, flavanone 3-hydroxylase, cytochrome P450 flavonoid monooxygenase, and flavonol synthase. In summary, apigenin, luteolin and quercetin have been successfully biosynthesized in microorganisms. Future investigations into the biosynthetic pathways of these flavonoids produced by Polygonaceae endophytes may enhance the yields of flavonoids in engineered strains.

### 5.4 Biosynthesis of other metabolites

Endophytes derived from Polygonaceae plants produce metabolites such as gallic acid, 3-phenyllactic acid, and caffeic acid, which exhibit notable antioxidant and antimicrobial properties. These compounds possess significant applications in the food, pharmaceutical, and cosmetic industries. In response to the increasing demand, research efforts have concentrated on their synthesis using engineered strains of *E*. *coli* or *S*. *cerevisiae*.

Key enzymes involved in the biosynthesis of gallic acid include 3-dehydroshikimate (3-DHS) dehydratase and 4-hydroxybenzoate hydroxylase. The expression of 3-DHS dehydratase (*quiC*) and a mutated variant of 4-hydroxybenzoate hydroxylase (*pobA*) in *E*. *coli* has enabled the production of gallic acid (Guo et al., 2024). Following the optimization of metabolic flux and enhancement of the shikimate pathway, the engineered *E*. *coli* strain achieved a yield of 51.57 g/L during fed-batch fermentation.

3-Phenyllactic acid can be synthesized by lactic acid bacteria utilizing phenylalanine as a substrate, with the catalysis of aminotransferase and dehydrogenase (Rajanikar et al., 2021). During the production of 3-phenyllactic acid, dehydrogenase consumes NADH, necessitating the addition of formate dehydrogenase (FDH) for NADH regeneration (Zheng et al., 2015). Zhao et al. (2018) expressed L-amino acid deaminase, D-2-hydroxyisocaproate dehydrogenase, and FDH in *E*. *coli*, resulting in a strain with a high conversion rate of 81.3% for the transformation of L-phenylalanine into 3-phenyllactic acid (Zhao et al., 2018). The yield of 3-phenyllactic acid reached 121 mM (20.11 g/L).

The biosynthetic pathway of caffeic acid involves several key enzymes, including PAL, TAL, C4H, CPR, coumarate 3-hydroxylase (C3H), and 4-hydroxyphenylacetate 3-hydroxylase (4HPA3H) (Li et al., 2020; 2025). Li et al. (2020) introduced C3H and CPR1 into *S*. *cerevisiae*, resulting in a strain that produced caffeic acid at a yield of 18.131 mg/L. Liu et al. (2019) introduced the TAL and 4HPA3H genes, *hpaB* and *hpaC*, into *S*. *cerevisiae*, enabling the strain to produce 289.4 mg/L of caffeic acid. Recognizing the crucial role of cofactors play crucial role in the synthesis of caffeic acid, Chen et al. (2022c) engineered the recycling and supply of the cofactors FADH_2_, S-adenosyl-L-methionine, and NADPH in *S*. *cerevisiae*, resulting in a final yield of caffeic acid reaching 5.5 g/L. In *E. coli*, Wang et al. (2023) constructed a strain expressing TAL and HpaBC, achieving a caffeic acid yield of 234.7 mg/L. Furthermore, by knocking out genes in competing pathways and overexpressing the FAD synthesis gene and the transporter gene in the engineered strain, the yield of caffeic acid reached 7.92 g/L (Wang et al., 2023).

In summary, significant advancements have been made in the microbial biosynthesis of active compounds with attractive yields. Although these compounds can be produced by endophytes from the Polygonaceae family, the pathway genes expressed in heterologous microorganisms for constructing engineered strains are rarely derived from Polygonaceae endophytes. Current studies commonly utilized genes from plants due to the limited understanding of synthetic pathways in microorganisms. Since gene source is crucial for the production of metabolites in heterologous hosts, and endophytes are microorganisms whose pathway genes may exhibit better compatibility to microbial hosts over plants. Investigating the undeciphered biosynthesis pathways of metabolites in endophytes from Polygonaceae plants will provide promising avenues for the biosynthesis of secondary metabolites or natural products in microbial cell factories, and facilitate the utilization of natural resources.

## 6 Conclusion and perspectives

This article reviews the diversity of endophytes in Polygonaceae plants, emphasizing their crucial roles in host plants, and summarizes various biological activities and the heterologous production of secondary metabolites derived from these endophytes. Among the four genera of Polygonaceae reviewed, a greater number of endophytic fungi were isolated and identified compared to endophytic bacteria. The genus *Fusarium* was found to be the most prevalent among the endophytic fungi, while *Bacillus* was identified as the most common genus of endophytic bacteria. Endophytes exhibit potential for enhancing yield and quality in cultivated Polygonaceae species. Furthermore, endophytes in Polygonaceae plants can produce a variety of high-value medicinal compounds with antimicrobial, antioxidant, and anticancer activities. The ability to produce bioactive substances indicates the presence of natural product biosynthetic pathways in Polygonaceae endophytes. Consequently, endophytes from Polygonaceae plants demonstrate promising prospects for utilization in natural product biosynthesis.

The Polygonaceae family constitutes a large group of plants with numerous genera. This review, however, is limited to a select subset of species within four of these genera, and endophytes from other genera are not addressed. For instance, plants belonging to the genus *Pleuropterus* are crucial representatives of the Polygonaceae family due to their pharmacological importance in traditional Chinese medicine, yet their endophytic communities remain largely unexplored. The underexplored endophytes in Polygonaceae plants may possess unique functional potentials, necessitating future investigation. Recent studies have increasingly focused on exploring the molecular mechanisms underlying endophyte-host interactions (Chen et al., 2022b; Ding et al., 2022), aiming to bridge the gap regarding the influence of these interactions on the regulatory mechanisms governing metabolite biosynthesis. Related studies have explored the mechanisms underlying endophyte-mediated growth promotion and stress tolerance enhancement in Polygonaceae plants. A deeper understanding of the biosynthetic pathways of secondary metabolites produced by endophytes in these plants should be pursued through multi-omics and synthetic biology approaches.

**FIGURE 3:**
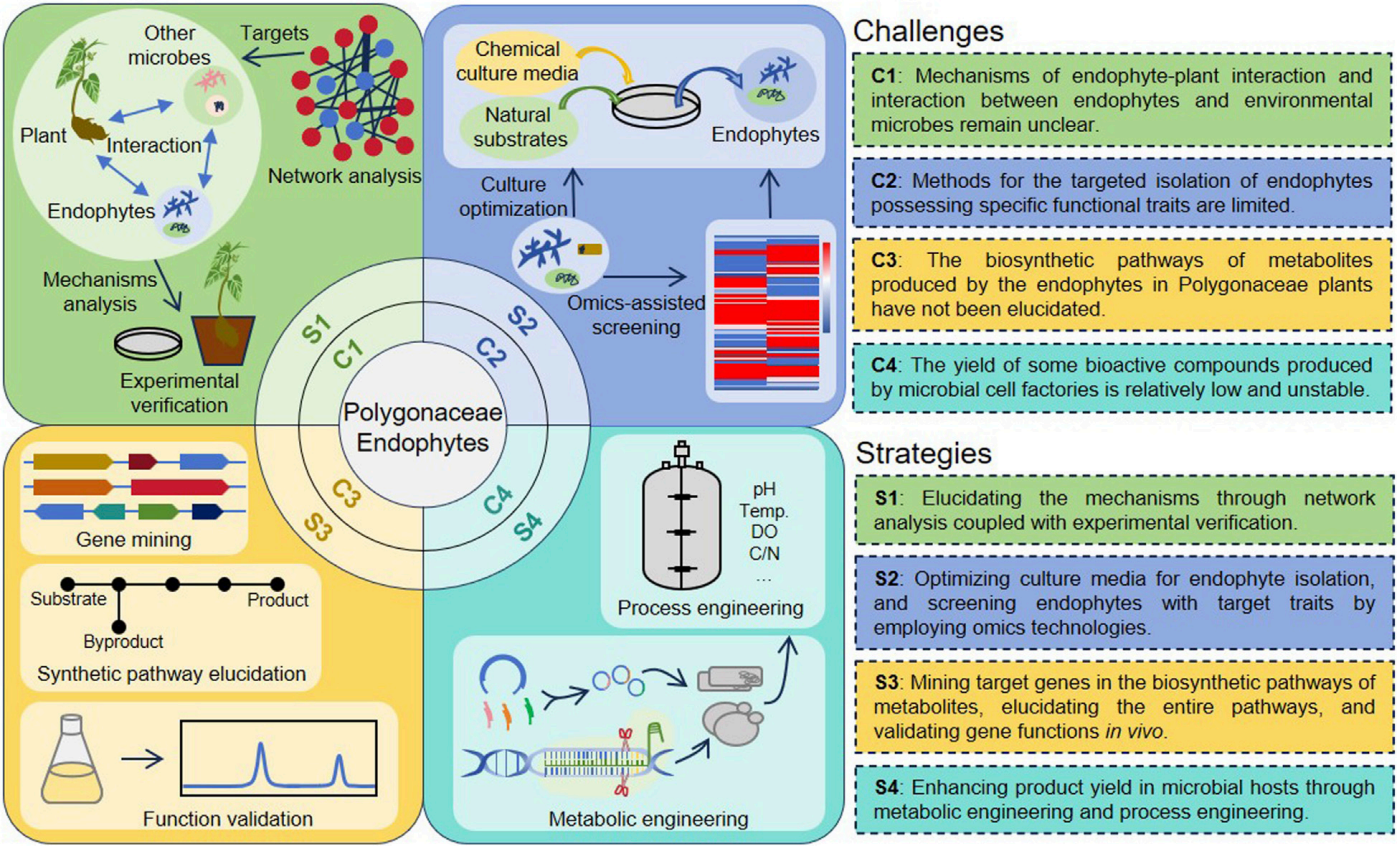
Challenges and future research directions in Polygonaceae endophytes.

Despite numerous studies on Polygonaceae endophytes, practical applications continue to face persistent challenges (Figure 3): The interactions between endophytes and plants, as well as among microorganisms, critically influence endophytic colonization and functionality within the host, however, the mechanisms underlying these interactions remain poorly understood. Although the diversity of endophyte species is exceedingly high, relatively few methods exist for the efficient isolation of endophytes with desired properties. A significant number of endophytes have been identified using second-generation sequencing technology, yet many remain unculturable under laboratory conditions. While Polygonaceae plants can produce a variety of natural products, their biosynthetic pathways have not been elucidated further. Challenges such as low yield and instability of production performance in Polygonaceae endophytes remain unresolved.

To address these challenges, several strategies are proposed (Figure 3). Using network analysis and experimental verification using various well-established methods to elucidate the mechanisms underlying the interactions between endophytes and plants, as well as with other microorganisms. A combined approach that utilizes multiple omics technologies should be developed to efficiently identify target endophytes with specific characteristics. Additionally, innovative culture methods for endophytes are essential for advancing their applications. Therefore, refining culture methods is critical for broadening the spectrum of culturable endophytes, which will facilitate the comprehensive utilization of endophytic resources. Furthermore, bioinformatics and genetic techniques will aid in elucidating the natural product biosynthetic pathways in Polygonaceae endophytes. Future efforts should focus on the metabolic engineering of endophytes or the heterologous expression of pathway genes to achieve high-level production of metabolites.
